# A DArT marker-based linkage map for wild potato *Solanum bulbocastanum* facilitates structural comparisons between *Solanum* A and B genomes

**DOI:** 10.1186/s12863-014-0123-6

**Published:** 2014-11-18

**Authors:** Massimo Iorizzo, Liangliang Gao, Harpartap Mann, Alessandra Traini, Maria Luisa Chiusano, Andrzej Kilian, Riccardo Aversano, Domenico Carputo, James M Bradeen

**Affiliations:** Department of Horticulture, University of Wisconsin, 1575 Linden Drive, Madison, WI 53706 USA; Department of Plant Pathology, University of Minnesota, 495 Borlaug Hall/1991 Upper Buford Circle, St. Paul, MN 55108 USA; Department of Agricultural Sciences, University of Naples Federico II, Via Università 100, 80055 Portici, Italy; Wellcome Trust Sanger Institute, Wellcome Trust Genome Campus, Hinxton, Cambridgeshire CB10 1SA, London, United Kingdom; Diversity Arrays Technology, Pty. Ltd., University of Canberra, Kirinari Street, Bruce ACT 2617 Canberra, Australia; Stakman-Borlaug Center for Sustainable Plant Health, 495 Borlaug Hall/1991 Upper Buford Circle, St. Paul, MN 55108 USA

**Keywords:** *S. bulbocastanum*, Linkage map, DArT markers, *S. tuberosum*, *S. lycopersicum*, Comparative genomics

## Abstract

**Background:**

Wild potato *Solanum bulbocastanum* is a rich source of genetic resistance against a variety of pathogens. It belongs to a taxonomic group of wild potato species sexually isolated from cultivated potato. Consistent with genetic isolation, previous studies suggested that the genome of *S. bulbocastanum* (B genome) is structurally distinct from that of cultivated potato (A genome). However, the genome architecture of the species remains largely uncharacterized. The current study employed Diversity Arrays Technology (DArT) to generate a linkage map for *S. bulbocastanum* and compare its genome architecture with those of potato and tomato.

**Results:**

Two *S. bulbocastanum* parental linkage maps comprising 458 and 138 DArT markers were constructed. The integrated map comprises 401 non-redundant markers distributed across 12 linkage groups for a total length of 645 cM. Sequencing and alignment of DArT clones to reference physical maps from tomato and cultivated potato allowed direct comparison of marker orders between species. A total of nine genomic segments informative in comparative genomic studies were identified. Seven genome rearrangements correspond to previously-reported structural changes that have occurred since the speciation of tomato and potato. We also identified two *S. bulbocastanum* genomic regions that differ from cultivated potato, suggesting possible chromosome divergence between *Solanum* A and B genomes.

**Conclusions:**

The linkage map developed here is the first medium density map of *S. bulbocastanum* and will assist mapping of agronomical genes and QTLs. The structural comparison with potato and tomato physical maps is the first genome wide comparison between *Solanum* A and B genomes and establishes a foundation for further investigation of B genome-specific structural chromosome rearrangements.

**Electronic supplementary material:**

The online version of this article (doi:10.1186/s12863-014-0123-6) contains supplementary material, which is available to authorized users.

## Background

The genus *Solanum* includes agronomically important plants such as potato (*S. tuberosum*), tomato (*S. lycopersicum*) and eggplant (*S. melongena*). Although distinct in terms of morphology and culinary utility, molecular dating suggests that potato and tomato are closely related species, having diverged from a common ancestor 7.3 million years ago [1]. Today, the potato clade comprises approximately 200 tuber-bearing *Solanum* species, including the cultivated potato and wild relatives native to South, Central, and North America. These wild species are potentially rich sources of genes for the improvement of the cultivated potato.

As a tool for the utilization of wild crop relatives to improve cultivated species, Harlan and Wet [2] developed the Gene Pool Concept with the primary, secondary, and tertiary gene pools reflecting crossability of wild species with cultivated crop plants. Because they are sexually compatible with cultivated species, germplasm in the primary and secondary gene pools can be directly utilized for crop improvement. In contrast, tertiary gene pool species are sexually isolated from cultivated crops and the genes they harbor cannot be accessed using traditional breeding approaches. Among potato species, the Endosperm Balance Number [3] predicts the crossability of species, with the cultivated potato assigned an EBN4 and most secondary gene pool species assigned to EBN2. Manipulation of potato ploidy levels can enable cross compatibility between secondary genepool, EBN2 species and the EBN4 cultivated potato, allowing incorporation of genes from wild species for crop improvement. In contrast, about 20 wild potato species are sexually isolated from cultivated potato and comprise the tertiary gene pool for *S. tuberosum.* These species predominantly have an EBN1 and post-zygotic barriers have significantly precluded widespread use of EBN1 species in potato breeding.

Among EBN1 potato species, the diploid (*2n* = 2x = 24) *S. bulbocastanum,* a native of southern Mexico and Guatemala, has long been of interest to potato breeders. The species is a famous source of resistance to late blight disease [4-9] and is a documented source of nematode resistance [10]. Like other tertiary gene pool species, however, *S. bulbocastanum* is sexually isolated from cultivated potato [4,11]. Although costly and time consuming, late blight resistance genes have been transferred from *S. bulbocastanum* to the cultivated potato genome using multi-species bridge crossing, somatic hybridization, and transgenic techniques [12-17]. Morphologically, *S. bulbocastanum* is one of the most distinct tuber-bearing potato species [18] and both morphology and molecular data indicate that *S. bulbocastanum* is phylogenetically distinct from cultivated potato [19,20]. Consistent with sexual incompatibility and phylogenetic uniqueness, cytological observations have led to conclusions that the genome of *S. bulbocastanum* (B genome) is structurally distinct from that of cultivated potato (A genome) and those of many other wild potato relatives (A, C, D and P genomes) [18,21].

Previous genetic mapping studies have provided valuable starting points for discovering and documenting major genome structural rearrangements that occurred since the potato and tomato genomes diverged from a common ancestor [22-25]. Cytological and genomics analyses demonstrated that tomato and potato are differentiated by a series of whole arm inversions of chromosomes 2, 5, 6, 9, 10, 11 and 12 (Table 1) [26-30]. To date, no study has explicitly compared the organization of the proposed A and B *Solanum* genomes using DNA sequence technologies. Some genetic or genomic studies have been conducted in *S. bulbocastanum* [10,31]. However, the genome architecture of *S. bulbocastanum* remains largely uncharacterized, limiting the application of comparative genomics studies between *S. bulbocastanum* and other *Solanum* species.

**Table 1:** **Overview of chromosomal rearrangements between the potato and tomato genomes based on comparative cytological and genetic mapping**

**Chromosome (potato)**	**Tomato**	**Citation** ^**1**^
2	2 L inversion	a
3	Translocation	b
5	5S inversion	a, c, d, e, f
6	6S inversion	a, g
9	9S inversion	a, c, d, f
10	10 L inversion	a, c, d, f
11	11S inversion	a, c, f
12	12S inversion	a, c, f

^1^a: TG Consortium [30]; b: Sharma *et al.* [27]; c: Tanksley *et al.* [23]; d: Bonierbale *et al.* [22]; e: Livingstone *et al.* [24]; f: Szinay *et al.* [28]; g: Iovene *et al.* [29].

Diversity Array Technology (DArT, http://www.diversityarrays.com) is a community-based molecular marker technology that allows high-throughput and cost-effective genotyping of target species, without relying on prior genome sequence information. DArT involves the preparation of an array of individualized clones from a genomic representation, generated from amplified restriction fragments [32,33]. The technology has been successfully utilized in various species including Arabidopsis [34], wheat [35], barley [32], and potatoes [36,37].

Sliwka *et al*. [36] utilized DArT technology to genotype a mapping population of *Solanum* x *michoacanum* to map the late blight resistance gene *Rpi-mch1*. The study generated a linkage map consisting of 798 DArT markers. In a separate study, Sliwka *et al*. [37] mapped a second late blight resistance gene *Rpi-rzc1* (derived from *Solanum ruiz-ceballosii*) to chromosome 10 using DArT markers and sequence specific PCR markers. Our group pioneered the development of a DArT platform for genotyping EBN1 tertiary genepool potato species, including *S. bulbocastanum* [38]*.* In this study, we employed this DArT array to develop a linkage map for *S. bulbocastanum.* The generation of medium density genome-wide linkage maps for this species, sequencing of mapped DArT probes, and alignment of DArT sequences to reference sequences [39] allowed us to compare genome structures between the B genome wild potato and the genomes of cultivated potato and tomato genomes [30,40].

## Results and discussion

### Linkage map generation

*Solanum bulbocastanum* is a highly heterozygous diploid species with up to four alleles per marker locus segregating in an F_1_ population. This precludes traditional mapping strategies. Instead, we applied the pseudo-testcross strategy [41], generating two parental linkage maps (one for parent PT29 and one for parent G15) and one integrated linkage map.

For mapping parent PT29, a total of 458 DArT markers were integrated into a linkage map comprising 12 linkage groups (LGs), as expected (Table 2, Additional file 1: Figure S1). This map covers a genetic distance of 620.1 centimorgan (cM). However, 156 (34%) markers mapped to an identical location (at 0.0 cM), resulting in 302 (66%) uniquely positioned markers or approximately 0.5 markers per cM. In total 203 markers (44.3%) mapped in PT29 aligned to a unique location on the potato and tomato reference genome sequences, allowing us to anchor the 12 LGs to corresponding chromosomes. Overall, PT29 LGs corresponding to chromosomes 1, 5 and 8 had few markers.

**Table 2:** **Summary of the** ***S. bulbocastanum*** **linkage maps including parental maps (PT29 and G15) and the consensus map**

**PotatoChr.**	**PT29**				**G15**				**Consensus**			
	**# LGs**	**# markers**	**# unique** ^**1**^	**Length (cM)**	**# LGs**	**# markers** ^**2**^	**# unique**	**Length (cM)**	**# LGs**	**# markers**	**# unique**	**Length (cM)**
**1**	1	19	19	27.4	1	10	10	22.5	1	49	41	49.1
**2**	1	58	40	52.6	1	19	19	50.7	1	98	53	48.3
**3**	1	59	43	44.1	2	12 + 2	12 + 2	40.2 + 11.8	1	73	49	36.5
**4**	1	75	53	94.3	2	21 + 3	21 + 3	40.7 + 12.7	1	103	67	83.7
**5**	1	12	12	4.8	2	2 + 3	2 + 3	1.2 + 8.8	1	22	20	40.7
**6**	1	86	38	47.4	1	5	5	22.5	1	83	40	45.6
**7**	1	24	20	58.7	1	8	8	50.7	1	36	30	58.4
**8**	1	17	12	50.9	3	4 + 3 + 2	4 + 3 + 2	17.7 + 39.1 + 9.1	1	26	19	51.4
**9**	1	32	18	60.6	2	6 + 6	6 + 6	22.3 + 42.7	1	48	25	60.4
**10**	1	23	9	61.5	2	10 + 7	10 + 7	29.2 + 49.5	1	23	9	61.5
**11**	1	33	20	50.2	2	4 + 6	4 + 3	20.6 + 8.2	1	39	22	40.2
**12**	1	20	18	67.8	1	5	5	28.4	1	31	26	69.1
**Tot.**	12	458	302	620.1	20	138	135	529.01	12	631	401	644.8

^1^mapped to a unique location.

^2^Numbers separated by “+” describe multiple LGs in the *S. bulbocastanum* parental maps corresponding to the same *S. tuberosum* chromosome.

For mapping parent G15, a total of 138 DArT markers were integrated into a linkage map comprising 20 LGs, substantially exceeding the expected 12 LGs (Table 2, Additional file 2: Figure S2). The G15 linkage map covers a genetic distance of 529 cM. Three markers mapped to an identical location (at 0.0 cM) resulting in 135 (98%) uniquely positioned markers with 0.27 markers per cM. The comparatively small number of markers integrated into the G15 map (compared to the PT29 map) is likely due to the fact that PT29, but not G15, was a prominent contributor of features on the potato DArT array. Out of 138 markers incorporated into the G15 parental linkage map, 90 (65%) markers aligned to a unique location on the potato and tomato reference genome sequences, allowing anchorage of all 20 LGs to corresponding chromosomes.

The integrated *S. bulbocastanum* linkage map comprises 12 LGs with a total of 631 markers, 401 of which are uniquely positioned (64%) (Table 2, Additional file 3: Figure S3). The integrated map spans a total genetic distance of 644.9 cM, averaging 0.62 unique loci per cM. The LG corresponding to potato chromosome 4 is the largest, comprising 103 DArT markers spanning 83.7 cM. To map root-knot nematode resistance (*Rmc1*) from *S. bulbocastanum*, Brown *et al*. [10] developed a restriction fragment length polymorphism (RFLP) *S. bulbocastanum* linkage map using a mapping population derived from somatic hybrids between the wild species and cultivated tetraploid potato. A *S. bulbocastanum* linkage map comprising 48 RFLP markers (belonging to 12 linkage groups) was generated and the locus *Rmc1* was mapped. Several linkage maps using a wide range of molecular markers have been developed for *S. tuberosum* and others relative species [42]. The integrated *S. bulbocastanum* DArT marker map developed in the current study represents a greater than 10-fold increase in marker density compared to the only previously available genetic map for *S. bulbocastanum* [10].

### Comparative analysis between the *S. bulbocastanum* genetic map and the potato and tomato physical maps

Early comparison of low resolution RFLP linkage maps revealed general conservation of marker order along nine of the 12 chromosomes of potato and tomato with three chromosomes displaying intra-chromosomal, paracentric inversions that structurally distinguished the two genomes [22]. Subsequent increases in marker density and refinement of linkage maps confirmed and expanded these early observations [23,43] and sequencing of the potato [40] and tomato [30] genomes allowed direct comparisons. In total, sequence analysis identified nine large inversions and numerous small scale inversions that structurally differentiate the potato and tomato genomes [30]. These changes in chromosome structure have accumulated since divergence of the potato and tomato lineages from a common ancestor approximately 7.3 million years ago [1].

Over that same period of time, the potato clade has diversified to encompass approximately 200 extant tuber bearing *Solanum* species. Numerous factors including physical separation and sexual isolation due to differences in ploidy and EBN have facilitated morphological and phylogenetic diversification amongst potato species. *Solanum bulbocastanum,* the focus of the current study, is a diploid, EBN1 species that belongs to the tertiary gene pool for cultivated potato. The species is morphologically distinct, with simple, undivided leaves, and a star-shaped or stellate flower, a morphological characteristic considered to be evolutionarily primitive [19]. In contrast, the cultivated potato is an autotetraploid, 4EBN species with divided leaves and a fused or rotate corolla. Consistent with morphological classification, molecular data support clear phylogenetic distinction between EBN1 species, including *S. bulbocastanum,* and the cultivated potato [20].

Classical cytogenetics approaches led to postulations of structurally distinct genome configurations amongst potato species [21,44-47]. Various models and terminology were standardized by Matsubayashi [21]. Cultivated potato was designated as an A genome species and *S. bulbocastanum* was designated as a B genome species. Crosses between cultivated potato and *S. bulbocastaum* have consistently produced no viable progeny [11], precluding direct cytological observation of chromosome pairing behaviors between these species. Differences in A and B genome structures, where directly observable, include visible loops in paired chromosomes during pachytene. Hermsen and Ramanna [48] observed loops during pachytene in F1 progeny resulting from a cross between the A_1_ genome *S. verrucosum* and B genome *S. bulbocastanum,* concluding that the two genomes are structurally distinct, with differentiation consisting of a series of small scale structural differences. Phylogenies constructed based on DNA sequence of nitrate reductase [49] and Waxy [50] genes support differentiation of A and B genome species. Importantly, in allopolyploids comprising A and B genomes, these gene sequences remain distinct [49]. To date, no direct molecular comparison of potato A- and B-genome structures has been reported.

**Figure 1:**
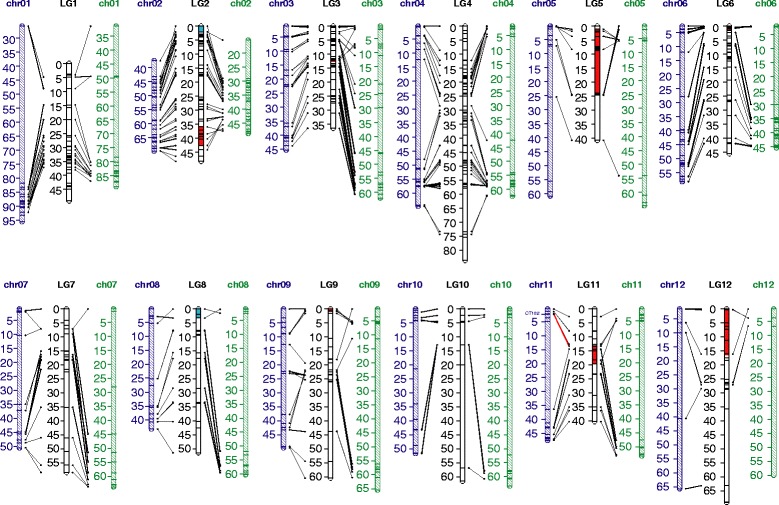
**Comparison of the** ***S. bulbocastanum*** **integrated genetic map with tomato and cultivated potato physical maps.** Dark blue: potato physical map (genome sequence); Green: tomato physical map (genome sequence); black: *S. bulbocastanum* genetic map (consensus DArT marker linkage map). On the *S. bulbocastanum* map, regions highlighted in red show higher collinearity to cultivated potato than to tomato. Regions of the *S. bulbocastanum* map highlighted in blue are segments with an arrangement distinct from that found in cultivated potato or tomato. These segments may be specific to *S. bulbocastanum* and other B genome *Solanum* species. Marker CT182 on potato Chr11 is linked to the Columbia root-knot nematode locus named *Rmc1* [10-52]. DArT marker 473601 highlighted with the red connection on *S. bublocastanum* LG11 represent the closest markers to CT182.

Previously we sequenced and characterized over 800 potato DArT array clones [38]. Of these, more than 500 were incorporated into the newly developed *S. bulbocastanum* genetic linkage maps described above. Alignment of DArT clone sequences to reference physical maps of tomato and cultivated potato [30,40] allowed direct comparison of DArT marker order on the *S. bulbocastanum* genetic map and corresponding genome regions of the potato and tomato sequences. In total, the *S. bulbocastanum* genetic maps represented over 86% of the total tomato and potato physical maps (Figure 1). Overall, we found a high degree of marker collinearity between *S. bulbocastanum* and potato and tomato (Figure 1, Additional file 4: Figure S4 and Additional file 5: Figure S5). In total nine genome structural changes between *S. bulbocastanum*, potato and tomato were identified (Table 3, Figure 1).

**Table 3:** **Summary of chromosomal rearrangements detected between** ***S. bulbocastanum*** **linkage map and the potato and tomato genomes**

**LG/chromosome (** ***S. bulbocastanum*** **)**	**Potato**	**Tomato** ^**1**^	**Position in tomato genome (Mb)**	
			**DArT** ^**2**^	**BAC FISH** ^**3**^
2	2S inversion	2S inversion	15.4 - 31.9	-
		**2 L inversion**	43.8 - 47.9	43.9 - 47.5
3		**3S translocation**	1.8 - 7.7	-
5		**5S inversion**	0.5 - 5.1	0.4 - 5.0
6		**6S inversion**	1.9 - 2.2	1.9 - 2.4
8	8S inversion	8S inversion	1.1 - 2.5	-
9		**9S inversion**	0.8 - 5.1	0.7 - 6.1
11		**11S inversion**	4.2 - 5.1	0.1 - 5.2
12		**12S inversion**	0.5 - 3.9	0.2 - 2.8

^1^Bold characters indicate chromosomal structural rearrangements previously identified between potato and tomato genomes as indicated in Table 1.

^2^identified based on alignment of DArT sequences (mapped in *S. bulbocastanum* consensus linkage map) flanking chromosomal rearrangements to tomato genome sequence.

^3^identified based on alignment of sequenced BAC clones to tomato genome sequence. The BAC clones flank chromosomal rearrangements used in cytological study by Peters *et al.* [26], Szinay *et al.* [28] and Iovene *et al.* [29].

Seven of the 9 rearrangements represent genome structure changes that have occurred since the initial speciation of the tomato and potato lineages as verified by cytological assays [26-30]. These rearrangements involve the long arm of chromosome 2(2 L) and the short arms of chromosome 5(5S), 6(6S), 9(9S), 11(11S) and 12(12S). In each instance, *S. bulbocastanum* shows high collinearity to the potato genome and rearrangement relative to the tomato genome. For example, a region of the *S. bulbocastanum* integrated map on LG3 spanning positions 11.6 to 12.2 cM is collinear with potato chr3S but rearranged relative to tomato chr3S. Specifically, DArT markers mapped in this region in *S. bulbocastanum* align to two disparate tomato chr3 positions: 1.8 Mb and 7.7 Mb (Table 3). Recently Sharma *et al*. [27] reported that this tomato 3S region contains an insertion that aligns to potato 3 L. The authors concluded that a translocation across the centromere differentiated potato and tomato chromosome 3. Our results are in agreement. Four markers covering the potato-tomato inversion on chromosome 10 (10 L) co-localized in *S. bulbocastanum* LG10 at position 12.8 cM. The lack of recombination between the four markers in our *S. bulbocastanum* F1 population precludes examination of the presence or absence of this rearrangement in the B genome (data not shown).

Collectively, our results suggest that B genome wild potato species share higher collinearity with cultivated potato than tomato, consistent with closer phylogenetic relationships between *S. bulbocastanum* and cultivated potato than between *S. bulbocastanum* and tomato [26].

Importantly, our study also suggests two rearrangements that differentiate *S. bulbocastanum* from both potato and tomato (Table 3). These comprise two independent inversions on *S. bulbocastanum* chromosome 2S and 8S (Figure 1). These segments span small genetic and physical distances (around 5–10 cM) and are located near telomere positions. Because these putative rearrangements are signified by relatively few markers, we cannot rule out errors in linkage mapping and greater marker saturation, expanded mapping populations, and other means of further validation by cytogenetic experiments are warranted. Given the phylogenetic distinction of *S. bulbocastanum* and potato, and cytological observations implying genomic structural differences between these species, we conclude accumulation of chromosomal structural variation in *S. bulbocastanum* relative to potato is not improbable.

To date no comparative mapping study has explicitly compared *Solanum* A- and B-genome species. The putative chromosome inversions we observed on *S. bulbocastanum* chromosomes 2(2S) and 8(S) could comprise a set of genomic structural changes discriminating between the *Solanum* A- and B-genomes. Expansion of mapping efforts, cytological study or whole genome sequencing of *S. bulbocastanum* and other B-genome *Solanum* species may confirm the legitimacy of these regions and may reveal other B-genome specific genomic segments. Since the original A vs. B genome hypotheses are based on low resolution cytological observations [21], we expected medium density linkage mapping in *S. bulbocastanum* to offer sufficient resolution to identify structural variations. Our approach demonstrates that molecular mapping with DArT markers followed by genomics analysis of mapped loci enabled identification of large-scale changes in chromosome structure, identifying seven major rearrangements that occurred since potato and tomato diverged.

Owing to their phylogenetic novelty, EBN1, B-genome *Solanum* species are likely sources of novel disease resistance and agronomic traits [51]. Documentation of predominant collinearity between A and B genome potato species and the validation of the DArT marker platform for comparative analyses provide new opportunities for potato improvement. The sequence of markers CT182, linked to Rmc1 locus [52] was used to identify the approximate location of this locus in the DArT map. A DarT markers (ID 473601) mapped at 13.3 cM of LG11, localized at position 2.38 Mb of potato Ch11, only 0.2 Mb apart from marker CT182 (2.40 Mb)(Figure 1). This paves the way for rapid mapping of genes underlying traits of interest and comparative approaches to gene mapping and cloning. Our ongoing efforts to isolate and map candidate disease resistance genes in *S. bulbocastanum* and other B-genome species [53,54] are likely to further this potential. Useful genes isolated from B-genome species can be transferred to potato as transgenes [17]. Somatic hybridization [15] and multi-species bridge crosses [12] provide non-transgenic approaches to introgress genes from B-genome species into cultivated potato. In these instances, marker aided selection (MAS) may provide a rapid and efficient means of generating improved commercially acceptable potato cultivars. The current study documents that the DArT marker platform could be useful for MAS approaches involving wild species germplasm.

## Conclusion

The first medium-density genome-wide linkage map for wild potato *S. bulbocastanum* was generated, demonstrating the utility of the DArT platform for genotyping wild potato species. Over 600 markers were integrated into the linkage maps, representing a greater than ten-fold increase in marker density compared to previously existing maps for the wild potato species. Sequencing and alignment of DArT clones to reference potato and tomato physical maps allowed a comparison of genetic and physical orders of the markers. Our results indicate that a majority of the markers are collinear between genetic and physical maps. Marker orders on *S. bulbocastanum* LGs show higher collinearity to the reference potato physical map than to the tomato physical map. Our research will assist comparative mapping of agronomical important genes or QTLs.

## Methods

### Plant material, DNA isolation, and DArT genotyping

Full-sib progeny seeds from a cross between wild potato *Solanum bulbocastanum* genotypes PT29 and G15 were planted at the University of Minnesota Plant Growth Facilities greenhouse (St. Paul, MN). Leaf tissue from seven week old plants was collected, frozen immediately in liquid nitrogen, and stored at −80°C for DNA extraction using a modified CTAB method [55].

In collaboration with the Diversity Arrays Technology, Pty. Ltd., a DArT array for wild potatoes (http://www.diversityarrays.com/) comprising over 20,000 features was constructed [37,38]. DNA samples from 92 F_1_ progeny of the cross PT29 X G15 together with the two parental lines (PT29 and G15) were genotyped using the DArT array and previously established protocols [31-33].

### Linkage map construction

We employed the pseudo-testcross strategy [41] to construct linkage maps. A total of 854 markers were coded into three marker classes. Markers that were heterozygous in PT29 but homozygous in G15 were coded into the lmxll class (490 markers). Markers that were homozygous in PT29 but heterozygous in G15 were coded into the nnxnp class (166 markers). Markers that were heterozygous in both parents were coded as hkxhk markers (198 markers).

Two parental maps were generated using lmxll (PT29 parental map) and nnxp (G15 parental map) markers, respectively. The regression mapping algorithm of JoinMap 4.1 (http://www.kyazma.nl/index.php/mc.JoinMap/) was used to generate the respective parental maps. Kosambi’s mapping function was used in calculating map distances. The two resulting parental maps were then merged into a composite map using anchor markers (hkxhk). Integrated map marker order was largely based on fixed marker orders from parental maps. In cases in which the two parental fixed marker orders could not be simultaneously satisfied, the marker order from PT29 was adopted.

### Comparison of marker order with potato and tomato physical maps

DArT clones polymorphic between the *S. bulbocastanum* mapping parents were subsequently sequenced [39] and the sequences were aligned to both potato and tomato genome sequences using GenomeThreader [56] with 70% minimal nucleotide coverage and sequence identity. Only uniquely aligned DArT clones (i.e., DArT sequences anchored to a single location in the reference genome sequence or to a cluster of identical sequences occupying a single contiguous location on the reference genome sequence) were used to compare physical and genetic maps. The comparative alignment information was summarized using a custom Perl script and visualized using MapChart v2.0 [57]. The list of markers, their location in the integrated map, the potato and tomato genomes was provided in Additional file 6.

### Availability of supporting data

The data set supporting the results of this article is included in Additional file 7 and available in the Genomic Survey Sequences (GSS) database under accession number KG961889 - KG963311.

## Additional files

Additional file 1: Figure S1
*Solanum bulbocastanum* PT29 genetic linkage map. A total of 458 DArT markers were mapped to 12 linkage groups representing 12 chromosomes. Additional file 2: Figure S2
*Solanum bulbocastanum* G15 genetic linkage map. A total of 138 DArT markers were mapped to 20 linkage groups representing 12 chromosomes. Additional file 3: Figure S3
*Solanum bulbocastanum* integrated genetic linkage map. A total of 631 DArT markers were mapped to 12 linkage groups representing 12 chromosomes. Additional file 4: Figure S4 Comparison of the *S. bulbocastanum* PT29 genetic map with tomato and cultivated potato physical maps. Dark blue: potato physical map (genome sequence); Green: tomato physical map (genome sequence); black: *S. bulbocastanum* genetic map (PT29 DArT marker map). On the *S. bulbocastanum* map, regions highlighted in red show higher collinearity to cultivated potato than to tomato. Regions of the *S. bulbocastanum* map highlighted in blue are segments with an arrangement distinct from that found in cultivated potato or tomato. These segments may be specific to *S. bulbocastanum* and other B genome *Solanum* species. Additional file 5: Figure S5 Comparison of the *S. bulbocastanum* G15 genetic map with tomato and cultivated potato physical maps. Dark blue: potato physical map (genome sequence); Green: tomato physical map (genome sequence); black: *S. bulbocastanum* genetic map (G15 DArT marker map). On the *S. bulbocastanum* map, regions highlighted in red show higher collinearity to cultivated potato than to tomato. Regions of the *S. bulbocastanum* map highlighted in blue are segments with an arrangement distinct from that found in cultivated potato or tomato. These segments may be specific to *S. bulbocastanum* and other B genome *Solanum* species. Additional file 6: Table S1 Information of the DArT markers mapped in the integrated linkage map. The marker ID, genetic location and potato and tomato genomic location were included. Additional file 7
**Sequences of the DArT markers used in this study.**
