# Supplementary figures and images for: A DArT marker-based linkage map for wild potato Solanum bulbocastanum facilitates structural comparisons between Solanum A and B genomes
Source: BMC Genet. 2014 Nov 18;15:123. doi: 10.1186/s12863-014-0123-6 (PMC4240817; doi:10.1186/s12863-014-0123-6)

PT29LG1

PT29LG2

PT29LG3

PT29LG4

PT29LG5

PT29LG6

PT29LG7

PT29LG8

PT29LG9

PT29LG10

PT29LG11

PT29LG12

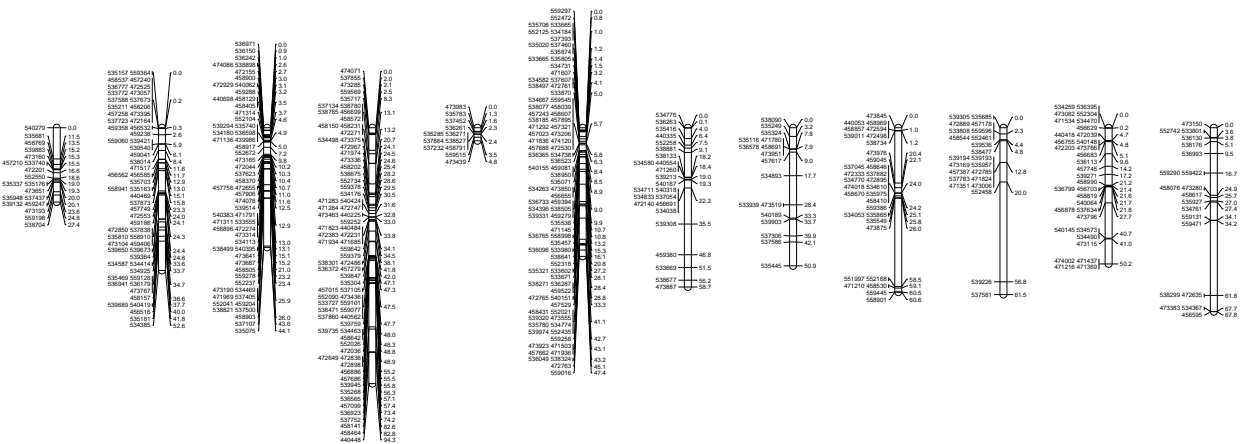

Supplement: Additional file 1: Figure S1 — Solanum bulbocastanum PT29 genetic linkage map. A total of 458 DArT markers were mapped to 12 linkage groups representing 12 chromosomes. [file 12863_2014_123_MOESM1_ESM.pdf]

G15LG1 G15LG2 G15LG3 G15LG4 G15LG5 G15LG6 G15LG7 G15LG8 G15LG9 G15LG10 G15LG11 G15LG12

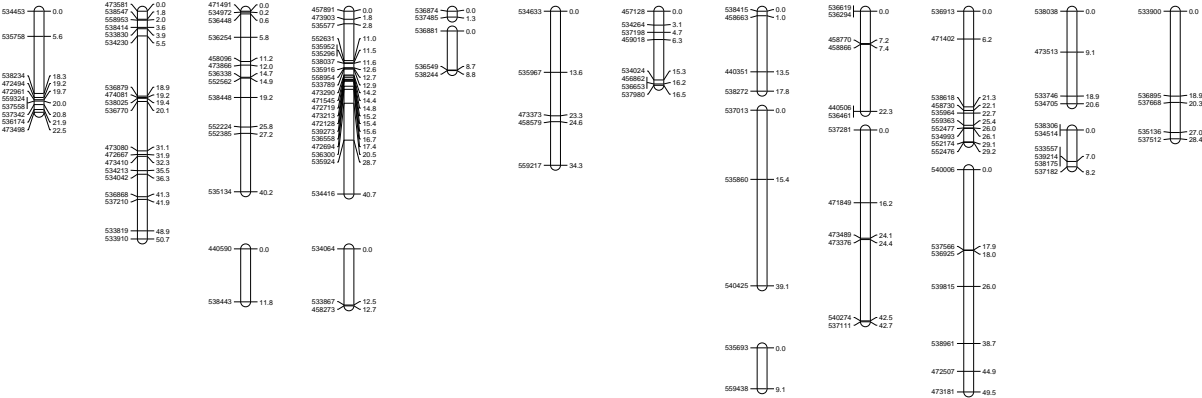

Supplement: Additional file 2: Figure S2 — Solanum bulbocastanum G15 genetic linkage map. A total of 138 DArT markers were mapped to 20 linkage groups representing 12 chromosomes. [file 12863_2014_123_MOESM2_ESM.pdf]

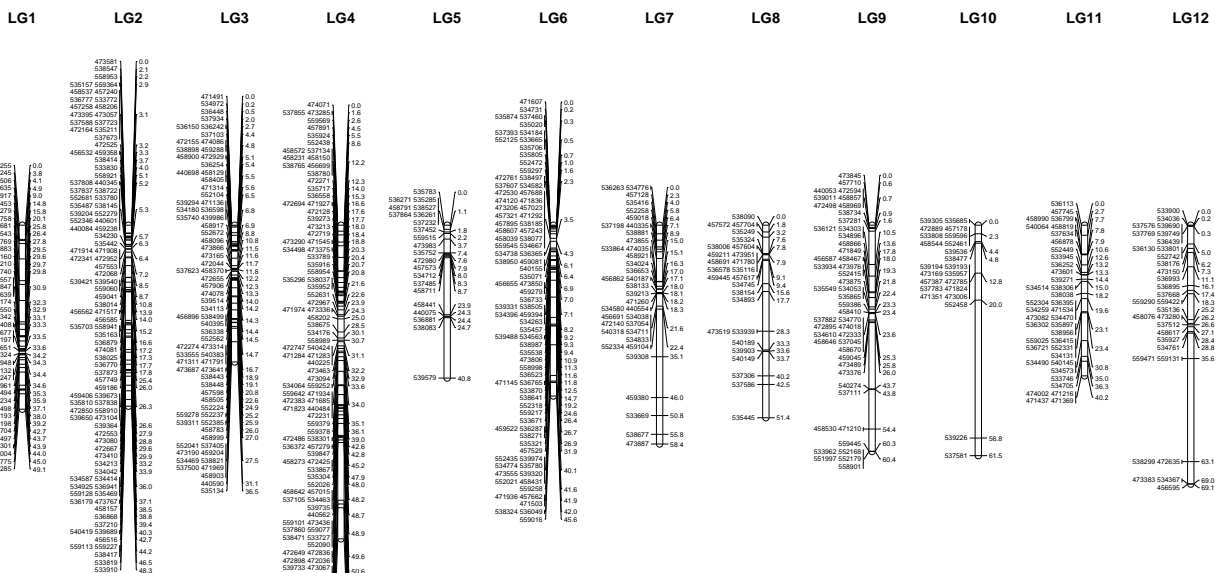

Supplement: Additional file 3: Figure S3 — Solanum bulbocastanum integrated genetic linkage map. A total of 631 DArT markers were mapped to 12 linkage groups representing 12 chromosomes. [file 12863_2014_123_MOESM3_ESM.pdf]

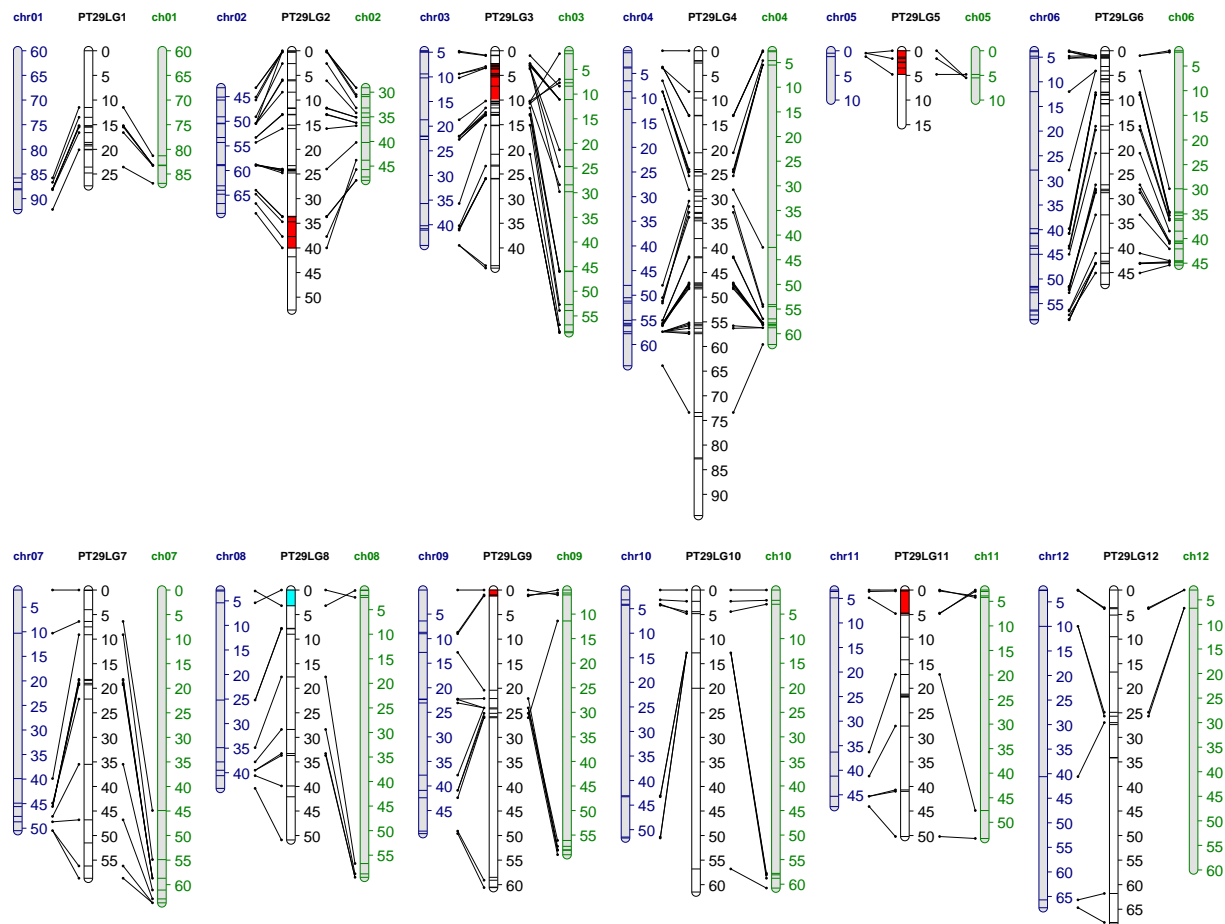

Supplement: Additional file 4: Figure S4 — Comparison of the S. bulbocastanum PT29 genetic map with tomato and cultivated potato physical maps. Dark blue: potato physical map (genome sequence); Green: tomato physical map (genome sequence); black: S. bulbocastanum genetic map (PT29 DArT marker map). On the S. bulbocastanum map, regions highlighted in red show higher collinearity to cultivated potato than to tomato. Regions of the S. bulbocastanum map highlighted in blue are segments with an arrangement distinct from that found in cultivated potato or tomato. These segments may be specific to S. bulbocastanum and other B genome Solanum species. [file 12863_2014_123_MOESM4_ESM.pdf]
