# Supplementary material for: A DArT marker-based linkage map for wild potato Solanum bulbocastanum facilitates structural comparisons between Solanum A and B genomes
Source: BMC Genet. 2014 Nov 18;15:123. doi: 10.1186/s12863-014-0123-6 (PMC4240817; doi:10.1186/s12863-014-0123-6)
Supplement: Additional file 5: Figure S5 — Comparison of the S. bulbocastanum G15 genetic map with tomato and cultivated potato physical maps. Dark blue: potato physical map (genome sequence); Green: tomato physical map (genome sequence); black: S. bulbocastanum genetic map (G15 DArT marker map). On the S. bulbocastanum map, regions highlighted in red show higher collinearity to cultivated potato than to tomato. Regions of the S. bulbocastanum map highlighted in blue are segments with an arrangement distinct from that found in cultivated potato or tomato. These segments may be specific to S. bulbocastanum and other B genome Solanum species. [file 12863_2014_123_MOESM5_ESM.pdf]

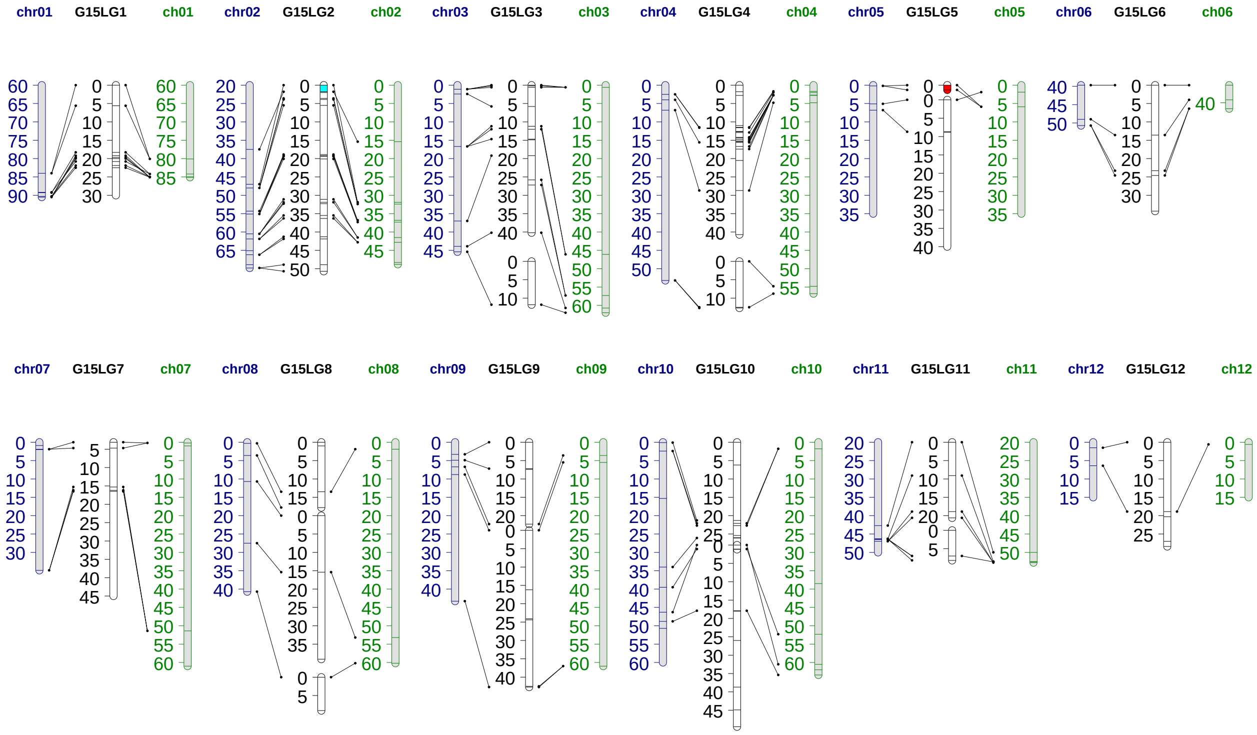
chr01
G15LG1
ch01
chr02
G15LG2
ch02
chr03
G15LG3
ch03
chr04
G15LG4
ch04
chr05
G15LG5
ch05
chr06
G15LG6
ch06
chr07
G15LG7
ch07
chr08
G15LG8
ch08
chr09
G15LG9
ch09
chr10
G15LG10
ch10
chr11
G15LG11
ch11
chr12
G15LG12
ch12
